# Formal comment on “Assessing the impact of the ‘one-child policy’ in China: A synthetic control approach”

**DOI:** 10.1371/journal.pone.0222705

**Published:** 2019-11-06

**Authors:** Daniel Goodkind

**Affiliations:** Independent Researcher, Arlington, VA, United States of America; University of Louvain, BELGIUM

## Abstract

For nearly half a century, parents in China have faced compulsory quotas allowing them to have no more than one or two children. A great debate in recent years over the impact of this program on China’s population continues in *PLOS ONE* with the publication of Gietel-Basten et al. (2019). The core question concerns how much higher China’s birth rates might have been had birth quotas not been enacted and enforced. Gietel-Basten et al. argue that the selection of such comparators in recent studies may reflect subjective choices. They profess to avoid such subjectivities by using what they present to be a more scientific, objective, and transparent statistical approach that calculates a weighted average of birth rates of countries with other characteristics similar to China’s. Yet the authors make subjective choices regarding the non-fertility characteristics used to form their comparators which leads to an underestimation of the impact of birth planning. Moreover, their visual presentation, which focuses on the two key sub-phases of the birth program, underrepresents its overall impact. Their comparators suggest that China’s population today would be just 15 million more had it not enacted any birth restrictions since 1970 (one percent above its current population) and that in the absence of one-child limits, which began in 1979, China’s population would be 70 million *less*. At the same time, the authors acknowledge that the one-child program has had numerous negative consequences. It seems fair to ask how such consequences could result if the program had no significant impact on childbearing decisions.

## Introduction

China began in 1970 to control the size of its population through compulsory birth quotas, an effort it saw as essential to its rise (and restoration) as a world power. The demographic impact of this now half-century implementation of one- or two-child quotas has inspired considerable debate. In a 2017 article published by *Demography*, I estimated that had China not imposed birth restrictions, its population would now be well more than half a billion larger and that by 2060 its population would be *one billion* larger [*1*]. These estimates were based on an alternate model of China’s fertility decline proposed by colleagues drawn from 16 countries with fertility rates close to China’s in 1970 [*2, 3*]. The publication of the article was immediately followed by objections to my findings that China’s official estimates of the impact of this program were plausible, findings that were at odds with claims by other observers [*4, 5*].

The publication of Gietel-Basten et al. [*6*] continues the debate on this subject. The first author, a co-author of an earlier commentary [*5*], is now joined by two new authors to present a numerical exercise to assess the impact of China’s birth program. They claim that their advanced statistical methods provide a more scientific and objective way of constructing comparators for what China’s birth rates would have been in the absence of policy intervention. In this comment, I contend that the authors made subjective decisions in setting up their comparators which lead to an underestimation of the impact of the birth planning program. Moreover, their visual presentation of results, which focuses on the marginal impacts of the two major sub-phases of the program, provides an incomplete assessment of the program’s overall impact. Gietel-Basten et al. conclude that the one-child policy phase of the birth program (1980–2015) did “not have a statistically significant impact,” an apparent erasure of the impact of that program from history.

## Deep background

Most everyone has heard of China’s “one-child policy.” And that may be part of the problem, because this phrase is a misnomer [*7, 8*]. It refers to the strictest five-year phase (1980–1984) of a broader half-century program since 1970 designed to control China’s population. During the other 45 years, at least half of parents (and sometimes all) were allowed to have two children, or even more, given a variety of exemptions [*7*, *8, 9, 10*]. What makes this program so unique is that, unlike the family planning programs of other countries designed to help parents have the number of children they wanted, China imposed a *birth planning* program under which the government specified how many they could have [*11, 12*].

Much of the debate in this line of research reflects professional differences of opinion about which era deserves evaluation. In my view, such evaluations should begin by considering the impact of the entire half century of birth planning, which has included both one- and two-child quotas as well as evolving enforcements of such quotas. Although the title of Gietel-Basten et al. focuses on “the one-child policy” era during which some portion of parents faced one-child limits (1980–2015) [*2, 5*], to their credit, they start by evaluating the impact of the two-child program during the 1970s prior to one-child restrictions.

## Subjective choices in the application of the synthetic cohort method

In order to assess the impact of China’s birth restrictions, the core question is how much different China’s birth rates might have been had it not enacted such restrictions at two key points in time (which Gietel-Basten et al. refer to as policy “shocks”)–the beginning of the two-child era of the 1970s and the start of the one-child era in 1979. The key demographic measure they examine is the total fertility rate (TFR), which indicates the number of births a woman would expect to have in her lifetime if the birth rates in a given year were held constant.

Gietel-Basten et al. suggest that estimates in studies which select such counterfactual birth rates based on a single country or a basket of countries [*1*, *2, 3, 8, 13*] may reflect subjective choices. They offer the synthetic cohort approach as a better method for choosing comparators for China’s birth rates. The procedure requires the identification of characteristics other than fertility which, after application of their statistical model, will determine which countries are most similar to China during a given time period. Based on the resulting weights for each country, the synthetic comparator will be the weighted averages of the TFRs for those countries fixed in each year forward and backward in time. The authors then rely on a pair of population projections (based on the TFRs for China and the synthetic comparator) to determine how much higher China’s population would have been without policy intervention.

The authors present their application of the synthetic cohort approach as more scientific, objective, and transparent than prior efforts to gauge the impact of China’s birth policies. I disagree with these claims, for reasons described below.

First, the initial birth policy shock considered by the authors is said by them to date from 1973, the year when the dominant policy during the 1970s was formally dubbed *later-longer-fewer* [*10*]. That program promoted later marriage, longer birth intervals, and fewer births, with quotas of two births in some areas (and quotas of three births in others). However, as later pointed out by Gietel-Basten et al. [*6*], the policy restrictions under that program began at least two years earlier. In fact, China broke with Marxist precepts around 1970 when Premier Zhou Enlai specified maximum population growth targets for 1975 [*11, 12*]. By delaying the start of program until 1973, the authors estimate of China’s population reduction in the 1970s due to that shock (85 million) is too low.

Second, Gietel-Basten et al. made subjective choices regarding which non-fertility variables to include in constructing their synthetic cohorts. Although GDP per capita and average years of schooling seem reasonable choices, their decision to include average childbearing age is questionable, given that this variable is so closely correlated with fertility rates (the variable which the synthetic method will construct). Their inclusion of the sex ratio at ages 0–4 is also questionable. The authors note that sex ratio imbalances among young adults may be relevant for adversely affecting the likelihood of marriage for the sex in greater supply (males vs. females). Although that may indeed be true, one wonders why they chose to use instead sex ratios at ages 0–4, an age grouping at least twenty years below the typical age at marriage.

Third, given the lack of data in many countries for the non-fertility variables they chose, the number of countries available for their synthetic cohort analysis was greatly reduced. The original dataset included 187 countries for which fertility estimates were available, but that number shrank to only 64 once all the countries with missing data were excluded. The supplementary tables reveal that the 64 remaining countries had TFRs on average more than a full birth lower than the 123 countries that were excluded from the analysis (see below). The exclusion of countries with higher fertility (which tend to have lower incomes), forces their model to construct comparators for China from countries with lower fertility and higher incomes. Indeed, Table 1 of Gietel-Basten et al. confirms that the average income levels of the 64 countries available for their analysis is considerably higher than that which China had during comparable periods. None of the robustness checks presented by the authors address the biases that result from the exclusion of 123 countries from their analysis.

### Comparison of Mean Total Fertility Rates in Countries Included and Excluded in Gietel-Basten et al.

**Table:** 

187 countries 64 countries included 123 countries excluded
Mean 4.33 3.60 4.71

Fourth, the country weightings themselves raise concerns. Although one would expect variation in the weighted composition of countries at different points in time, the weights they show are unstable. Consider the highest weighted countries for the three successive policy periods examined:

Pre-1973 (which they use to estimate the impact of the two-child era of the 1970s):

India 36.9%, Jordan 21.1%, Thailand 15.2% (their Table 2)

For the period 1973–1979 (which they use to estimate the impact of the one-child era starting in 1979):

South Korea 75.2%, Thailand 16.0%, India 4.1% (their Table 2)

For the period 1980–1991 (to estimate the impact of another policy tightening in 1991):

Greece, 68.3%, India, 31.2% (their Appendix Table A2; no other countries exceeded 1 percent)

For instance, India, which accounts for more than 30 percent of the weighted average in the first and third eras, only constitutes 4 percent in the second era.

More worrisome is how three quarters of the comparator for the period 1973–1979 (used to gauge the impact of the subsequent one-child era) could be drawn from South Korea alone, a country that was experiencing perhaps the most rapid economic development of any country in the world around that time, at least two decades ahead of China. South Korea also experienced one of the world’s most rapid fertility declines and currently has the world’s lowest birth rates. For every year of the resulting series (see the dotted line on their Fig 1), three quarters of the comparator is drawn from the exceptional experience of South Korea. Then, in a supplementary analysis of the impact of a later policy shock in 1991, South Korea disappears and is replaced by Greece, which, now accounts for 68% of the weighting.

## Graphical depictions do not reflect the full impact of China’ birth planning program

To their credit, Gietel-Basten et al. [*6*] differentiate between two important questions to assess the impact of the major sub-phases of China’s birth planning program. First, how much higher would China’s fertility (and population) be if it had not implemented its birth limitation program at all (neither two-child nor one-child quotas nor any related enforcements)? Second, how much higher would China’s fertility (and population) be if it had implemented just the two-child program in the 1970s and thereafter maintained that program rather than tightening to one-child quotas around 1979/1980? Yet the first (and most critical) question is not adequately addressed in their discussion or the accompanying graphics, and because of that their answer to the second is debatable.

The dashed line in their Fig 1 shows the birth rates (TFRs) indicated by their synthetic cohort analysis assuming China had not implemented birth planning at all. As expected, the dashed line is well above the solid line (China’s observed TFR)–the difference is highlighted by pink shading–which suggests that the two-child program of the 1970s had a substantial impact in reducing fertility. According to the text, the corresponding gap on their Fig 3 shows that China’s population by 1979 would have been 85 million higher in the absence of the two-child program (higher even than the estimate of 73 million based on the aforementioned 16-country comparator; see Goodkind, 2017, Table 2) [*1*].

Thereafter, however, the authors cut off the dashed line in 1979 as well as the pink-shaded section below it. If the authors looked to answer their first question regarding the impact of the overall birth program, there appears to be no justification for such truncation. The dashed line could have been easily extended beyond 1979 based on the country weights derived from their “pre-1973” synthetic cohort analysis. Indeed, they include a graphic showing that very extension in an Appendix (their Fig A5), with no accompanying pink shading of the area below it or calculation of the massive population reduction it implies. Based on a visual comparison of the TFR series on their Fig A5 with comparators I examined in my prior work [*1*], their synthetic comparator appears to suggest that China’s population today is at least 700 million lower due to the birth planning program, half of its current population.

**Fig 1:**
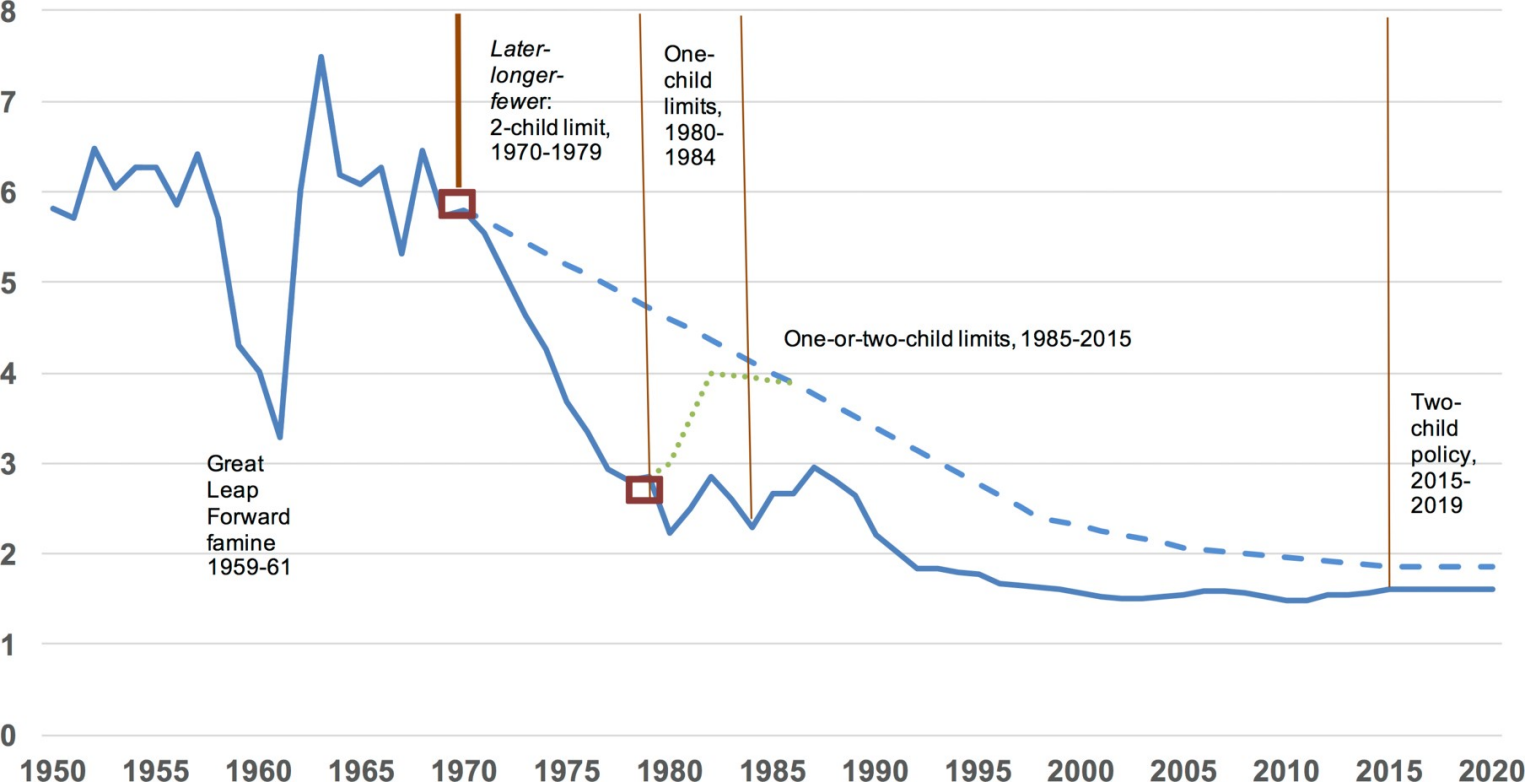
Births per woman in China, 1950–2020, and key phases of birth planning. Dashed blue line: Birth rates of 16 countries with fertility similar to China’s in 1970, Green dotted line: Proposed bounce in absence of one-child program, Solid blue line: China’s actual birth rates.

Instead of beginning with that summary calculation, and then attempting to divide the portions of it due to the two-child and one-child programs, respectively, the authors proceed to estimate after 1979 only the *marginal* impact of the second policy shock of one-child limits. Their Figs 1 and 3 suggest that this policy *raised* fertility *above* what their synthetic cohort comparator suggests it should have been (by up to half a birth in the 1980s), with China’s population by 2015 being 70 million *larger* because of it. The authors refer to this result as a “puzzling outcome,” which they attribute to a tendency for births to be overstated in official statistics. This explanation seems improbable given two decades of research findings that the penalties imposed under birth planning led to *understatement* of births by both parents and local officials [2, 11, 12]. Gietel-Basten et al. then reject their finding that the one-child program led to an increase in birth rates after robustness checks suggest that its impact was statistically insignificant (their Fig 2b).

The net overall impact of the two policy shocks as calculated by Gietel-Basten et al. implies that China’s population as of 2015 was only 1 percent smaller than would have been expected in the wake of the most forceful attempt to control population in human history (85 million minus 70 million divided by China’s population of 1.4 billion), none of which can be attributed to the one-child program.

## The Under-addressed question: What was the impact of China’s birth planning program *overall*?

An answer to the first question posited by Gietel-Basten et al.–what would China’s fertility have been like if it had not enacted any birth restrictions at all–requires consideration of a graphic similar to that shown here (my Fig 1). The solid line shows the historical pattern of birth rates (TFRs) in China from 1950 to 2020 (sources from [*9, 1*]). The dashed line, beginning with the upper red rectangle in 1970, shows the counterfactual TFRs based on the aforementioned 16-country comparator [*1,2,3*] through 2020. The gap between the dashed line and the solid line represents the impact of China’s birth planning policies overall.

In the 1970s, the gap is clearly due to the two-child program of *later-longer-fewer*. After 1979, the ongoing gap needs to be attributed either to the two-child program, the one-child program, or some combination of each. Yet Gietel-Basten et al. never address the substantial demographic implications of that ongoing gap because in 1979 they switch to a different counterfactual series beginning with the *lower* red rectangle. In essence, as noted earlier, after 1979 their approach measures only the *marginal* impact of the tightening to one-child quotas compared to preexisting two-child quotas, not the combined impact of both quotas that results in the ongoing gap.

Based on their conclusion that the one-child policy shock had a statistically insignificant impact, the ongoing gap after 1979 would presumably have to be fully attributed to the two-child program. Indeed, Fig 1 shows that the sharpest decline in China’s birth rates occurred in the 1970s *prior to* one-child quotas. After those tightened quotas, despite a saw-tooth pattern in the 1980s which mirrored high-low tides of mass sterilization campaigns [*11,12*], there was no appreciable decline in birth rates overall. What is puzzling then is why the strictest one-child phase of the most comprehensive program to control births in history was not followed by a decline in fertility rates. For over 20 years, the typical conclusion by demographers has been the same one repeated by Gietel-Basten et al. [*6*]: that the one-child program was both ineffective and unnecessary, in the sense that it did not lead to any further decline in birth rates compared to what had already occurred under the two-child quotas of the 1970s and would have otherwise been expected based on developmental factors.

But this standard explanation seems implausible given all we know about what was happening in China at the time. In the early 1980s, nearly one in four Chinese women was sterilized (one out of every 16 women in the world), abortion rates doubled, and strong opposition to one-child limits in rural areas, due in part to son preference, led China in the mid 1980s to allow a “1.5”-child exemption for rural parents whose first child was a daughter [*3, 14*]. Parents living in areas with fines that were double those of other areas tended to have 0.3–0.4 fewer births [*15, 16*]. Moreover, given that birth rates were falling everywhere else in Asia during the 1980s, it seems likely that they would have fallen in China as well, yet they did not. Counterintuitively, Fig 1 appears to imply that China enacted a *pro-natal* policy in the 1980s that propped birth rates up rather than the most anti-natal policy ever enacted.

The explanation for this counterintuitive finding is likely related to a fallacy resulting from historical determinism [*17*]. The historical timeline of birth rates in China shows the end result of everything that happened in China’s past, including its policy choices. It does *not* show us what would have happened in a parallel universe if China had not made the choices that it did at each step along the way.

There are several reasons to consider (as outlined below) that if China had *not* proceeded with tighter one-child restrictions in 1980 its birth rates would have bounced up temporarily and that its tightened restrictions *prevented the bounce that would have otherwise occurred*. Thus, much of the gap on Fig 1 after 1979 between the dashed and solid lines may be due to tightened one-child quotas and their enforcements. Given that Gietel-Basten et al. do not consider this hypothesis, [*1, 8*], which is directly related to China’s unique context and sequence of birth policy implementation, I briefly summarize the arguments below.

The first reason to expect that birth rates were poised to bounce is that the two-child program of the 1970s had caused fertility to fall so quickly. Thus, many young women during this decade who were prevented from having as many children as they wanted–say, in their 20s –would likely want to make up for that loss a decade later in their 30s. Second, the sharp fall in fertility in the 1970s occurred under enforcements that were effective under collectivized agriculture, where pregnancies were readily monitored (and birth quotas enforced) under communal farming [*9*]. When China began to dismantle the agricultural collectives in the late 1970s, it undermined those very same mechanisms of enforcement [*10, 11, 12*]. Third, the end of collectivization meant that parents could no longer rely on support from the government to take care of them in their old age. They would now fall back, at least in the short run, on a more traditional source of social security–their own children. Fourth, the one-child program was accompanied, ironically, by a loosening of the later-marriage restrictions that had been enacted in the 1970s. Thereafter, a pent-up demand for childbearing is suggested by the young adults who rushed to get married after 1980, with the average marriage age falling by three years [*9*]. What resulted was a sharp rise in first births that offset to some extent the sharp decline in second or later births caused by the new one-child program [*18*].

In my previous work to isolate the impact of one-child limits, I assumed that China’s TFR would have rebounded to 4 births per woman in the absence of those limits (shown by the green dotted line on Fig 1 [*1, 8*]) based on the experience of Taiwan, which had a TFR of 4.0 ten years after falling from the same TFR of 5.8 that China had in 1970. Some observers question whether China’s TFR could have bounced up by more than a full birth after 1979 (from a TFR of 2.8 to 4.0) given that so few countries have ever evinced a rebound of that magnitude [*4, 5*]. However, China’s unprecedented fertility decline in the 1970s occurred under an unprecedented two-child policy that no other country had ever attempted. Thus, no other country provides a comparative basis for evaluating how high China’s fertility might have bounced back after 1979 had the one-child program not been enacted. Gietel-Basten et al. [*6*] seem to acknowledge this qualification by asking in their conclusion whether “it is possible to create a ‘synthetic China’ at all?” In general, synthetic comparators for China based on socio-economic indicators may be well-justified in some cases. However, given China’s unique context of *birth policy implementation* amidst considerable political change, comparative measures from other countries cannot capture the dynamics necessary to measure the impact of one-child restrictions after 1979.

## Concluding questions

Not everyone may agree with my interpretation as outlined above. Yet to my knowledge, no other resolution of the central puzzle in Gietel-Basten et al. [*6*]– which is observed elsewhere in demographic literature–has yet been put forward. Along with their claim that the one-child program had a statistically insignificant impact on China’s population, their introduction and conclusion both feature a list of its “numerous negative … consequences” which are “impossible to ignore.” These include forced abortions and sterilizations, the distortion of child sex ratios due in part to sex-selective abortion, deliberate under-reporting of over-quota children in surveys, corruption and abuses in the birth planning system itself, as well as “long term psychological consequences of prioritizing one-child families.”[*6*] In addition to statistical evidence for such negative consequences, personal testimonies of affected parents are also readily available [*19, 20, 21*]. It seems fair to ask how can such negative consequences resulted from a program otherwise presumed to be statistically insignificant and, thus, apparently inconsequential for childbearing decisions? And if that program was so lacking in consequence for Chinese families, why did so many critics over the last four decades advocate against this program? [*2, 3, 5, 21*]
